# Antioxidant and Analgesic Effect of Melatonin Involving Sirtuin 1: A Randomised Pilot Clinical Study

**DOI:** 10.1111/jcmm.70605

**Published:** 2025-05-19

**Authors:** Serafina Perrone, Silvia Carloni, Serena Benedetti, Micheal Weiss, Lucia Marseglia, Valentina Dell'Orto, Virginia Beretta, Noemi Pappagallo, Marica Pagliarini, Maria Cristina Albertini, Patrizia Ambrogini, Walter Balduini, Angela Simona Montalto, Pietro Impellizzeri, Giuseppe Buonocore, Eloisa Gitto, Carmelo Romeo

**Affiliations:** ^1^ Neonatology Unit, Department of Medicine and Surgery, Pietro Barilla Children's Hospital University of Parma Parma Italy; ^2^ Department of Biomolecular Sciences University of Urbino Carlo Bo Urbino Italy; ^3^ Department of Pediatrics University of Florida Gainesville Florida USA; ^4^ Pediatric and Neonatal Intensive Care Unit University of Messina Messina Italy; ^5^ Department of Molecular Medicine and Development University of Siena Siena Italy

**Keywords:** analgesia, melatonin, miRNAs, oxidative stress, pain, sirtuin 1

## Abstract

Melatonin is a potent antioxidant molecule, and its analgesic effects have been observed in children. However, the underlying mechanisms of these effects have not yet been fully explored in clinical studies. We tested the hypothesis that melatonin reduces pain and oxidative stress involving the sirtuin pathway. Forty‐four children were randomly assigned to oral supplementation with melatonin or placebo before induction of anaesthesia for surgery. Plasma levels of 4‐hydroxynonenal (4‐HNE), melatonin, sirtuin 1 (SIRT1) and circulating miR‐34 and miR‐124a were analysed at T0 (pre‐hospitalisation), T1 (before surgery) and T2 (1 h after the end of the surgery). Melatonin decreased 4‐HNE and increased SIRT1 concentrations at T2 in supplemented children. Significant correlations were found between melatonin and pain score (*R* = −0.404), 4‐HNE and pain score (*R* = 0.44), melatonin and 4‐HNE (*R* = 0.42), 4‐HNE and SIRT1 (*R* = −0.43) and melatonin and SIRT1 (*R* = 0.41) at T2. Circulating miR‐34 and miR‐124a modulation were also observed. The reduction of oxidative stress and the modulation of circulating miR‐34 and miR‐124a, which target SIRT1 activity, suggest a novel pathway underlying melatonin's antioxidant and analgesic effects.

**Trial Registration:** ClinicalTrials.gov identifier: NCT06724432

## 1 Introduction

Melatonin (n‐acetyl‐5‐methoxytryptamine) is an endogenously produced indoleamine primarily formed by the pineal gland, which is a potent free radical scavenger, as well as an indirect antioxidant. Melatonin, as well as several of its metabolites, are highly effective direct free radical scavengers, with the ability to remove singlet oxygen (^1^O_2_), superoxide anion radical (O^2^), hydroperoxide (H_2_O_2_), hydroxyl radical (^•^OH) and the lipid peroxide radical (LOO^•^) [1, 2].

In addition to its well‐established antioxidant effects, melatonin has also been reported to exert analgesic properties [3]. Multiple preclinical studies have demonstrated its efficacy and safety in alleviating neuropathic and inflammatory pain in animal models [4, 5], findings that have supported its clinical use in a variety of pathological conditions, including perioperative settings [6, 7]. Although the exact mechanisms underlying melatonin's analgesic effects are not yet clearly understood, current evidence suggests involvement of β‐endorphins, opioid receptors, dopamine D_2_ receptors, MettaloThionein2 (MT_2_) and gamma‐Aminobutyric Acid (GABA A) receptors, and the nitric oxide (NO)–arginine pathway [8, 9, 10].

Pain is closely associated with oxidative stress (OS), often triggered by tissue injury, inflammation and the release of proinflammatory cytokines [11]. In neonates, early oral administration of melatonin has been shown to significantly reduce plasma levels of OS biomarkers such as total hydroperoxides and advanced oxidation protein products [12]. Elevated levels of these markers have been observed in neonates experiencing high pain scores, suggesting a close link between OS and pain [13]. After administration of exogenous melatonin, indeed, a significant reduction in lipid and protein peroxidation in the postoperative period has been reported in neonates [14] as well as analgesic adjuvant during ventilation [3].

Recent studies have highlighted the role of sirtuin 1 (SIRT1) in pain modulation, demonstrating its analgesic effects in both neuropathic and inflammatory pain conditions [15]. SIRT1 has emerged as a key regulator of redox balance and inflammation [16, 17], making it a plausible mediator of melatonin's combined antioxidant and anti‐nociceptive effects. Emerging evidence also points to the involvement of microRNAs (miRNAs), which are small non‐coding RNAs that fine‐tune gene expression, in modulating SIRT1 activity. Among several miRNAs, miR‐34 and miR‐124 have been shown to directly target SIRT1, suggesting a significant role in controlling its expression and functional impact [18], which may further contribute to the beneficial effects of melatonin.

In this prospective, randomised, double‐blind pilot study, we tested the hypothesis that melatonin reduces pain and oxidative stress involving the sirtuin pathway.

## 2 Methods

### 2.1 Recruitment and Randomization

The trial was registered and approved by the local Ethics Committee (Local No. 125222022) according to the Helsinki Declaration of 1964. Patients' legal guardians provided written informed consent. This pilot study was designed primarily to explore the potential effects of melatonin treatment in a surgical context. The total sample size of 50 patients was chosen based on practical considerations and consistency with similar pilot studies in the field.

Children between 3 and 5 years of age scheduled for elective surgery were prospectively enrolled at the Department of Paediatric Surgery, University Hospital of Messina, Italy, from January 2021 to June 2021. Inclusion criteria were the need for elective surgery in the morning time. Exclusion criteria were children with cerebral malformations and/or injuries or surgery in the afternoon or at night to eliminate conditions that could affect melatonin production. Children were also excluded in cases of withdrawal of informed consent, insufficient blood samples and haemolysis of the blood sample, as haemolysis interferes with the biochemical determination of the OS biomarkers.

When children were identified as eligible, if the parents consented, the children were randomly assigned to either Melatonin or the Control group (Figure S1).

Permuted block randomisation was performed by biostatistics using computerised sequences, and group assignments were provided in concealed opaque envelopes. Parents of participants were informed of their infant's group allocation at discharge.

### 2.2 Interventions

Melatonin (Dicoson, Dicofarm, Italy, 5 drops = 1 mg) was administered orally. The product is listed in the Register of Dietary Supplements on the Ministry of Health website (http://www.ministerosalute.it/alimenti/dietetica) and is classified with the following code: 943314283. This product is subject to the European Directive on Foodstuffs according to DL n. 169 of 21 May 2004, and not to the European Directive on Medicines 2001/20/EC transposed at the Italian level with D.L. n. 211 of 24 June 2003. Melatonin administration has a good safety profile, with no known adverse effects [14].

Approximately 1 h before induction of anaesthesia, patients received oral melatonin or placebo. Melatonin‐treated children received a single dose of oral melatonin 0.5 mg/kg (for a max 10 mg). Melatonin (Dicoson, Dicofarm, Italy) was prepared by a dedicated resident in a fixed volume of 5 mL by adding water to a syringe without a needle. Five per cent dextrose (placebo) was used to simulate the sweet taste of the melatonin formulation in the Control group.

The contents of the syringe were blindly administered to the patients by the attending nurse, who was not involved in the study.

Samples of 0.2 mL of plasma were collected at T0 (pre‐hospitalisation, 1 day before surgery), T1 (after anaesthesia, immediately before surgery) and T2 (at 1 h after the end of the surgery and awakening after anaesthesia), and biochemical analyses were performed.

### 2.3 Perioperative Management of Children, Analgesia and Anaesthesia

The induction of general anaesthesia was obtained in all children through bolus doses of 1 mg/kg over 20 s of intravenous propofol, followed by similar bolus doses of propofol until the patient was anaesthetised, and anaesthesia was maintained with inhaled sevoflurane.

Children were considered anaesthetised when they were asleep and unarousable, and the eyelash reflex disappeared. Thereafter, anaesthesia was maintained with inhaled sevoflurane.

At the end of the surgery, after returning to the ward, all patients were assessed for postoperative pain using the Face, Legs, Activity, Cry and Consolability (FLACC) scale [14]. If the FLACC score was greater than 3, the analgesic drug paracetamol was administered at the dose of 15 mg/kg every 6 h at most. Clinical and research personnel were unaware of the group assignments until the completion of data analysis. The number of doses of paracetamol administered to children of both treatment conditions within 48 h after surgery, even if discharged, was recorded.

### 2.4 Melatonin, 4‐HNE and SIRT1 Measurements

Melatonin plasma levels were assessed at each experimental time point (T0, T1, T2) using a competitive enzyme‐linked immunosorbent assay (cELISA) kit from Antibodies.com (A87093) according to the manufacturer's instructions. T1 and T2 plasma samples from melatonin‐treated children suspected of containing concentrations higher than the highest standard (500 pg/mL) were diluted 1:100 (v/v) with sample diluent prior to analysis. Colour development was monitored at 450 nm in a Thermo Scientific (MultiSkan FC) microplate reader, and a standard curve (range 7.813–500 pg/mL) was generated using a four‐parameter logistic (4‐PL) curve fit. The sensitivity of the assay was 4.688 pg/mL; the intra‐ and inter‐assay coefficients of variation were < 8% and < 10%, respectively.

4‐HNE as a marker of lipid peroxidation was measured to evaluate OS by using a cELISA kit from Antibodies.com (A86962). Plasma concentrations were calculated by reading the absorbance at 450 nm and referring to the standard curve (range 31.25–2000 pg/mL). The sensitivity of the assay was 18.75 pg/mL; the intra‐ and inter‐assay coefficients of variation were < 8% and < 10%, respectively.

SIRT1 was quantified using an ELISA kit from Invitrogen (EH427RB). Plasma samples were diluted 1:2 as indicated by the manufacturer before analysis. SIRT1 concentrations were calculated by absorbance reading at 450 nm and referring to the standard curve (range 1.23–300 ng/mL). The sensitivity of the assay was 1.23 ng/mL; the intra‐ and inter‐assay coefficients of variation were < 10% and < 12%, respectively.

### 2.5 Quantitative Real‐Time PCR for Mature MicroRNA Analysis

We performed microRNA analyses based on the quali‐quantitative plasma sample available (*N* = 3 at each time point, T0, T1 and T2). MicroRNAs (miR‐34 and miR‐124a) were isolated from plasma using the Norgen total RNA isolation kit [15]. The plasma microRNAs and spike‐in cel‐miR‐39 expressions were evaluated using the TaqMan miRNA assay. The TaqMan miRNA reverse transcription kit was used to reverse transcribe miRNAs. Subsequently, RT‐qPCR was performed in 20 μL of PCR mix containing 1 μL of 20× TaqMan miRNA assay, which contained PCR primers and probes (5′‐FAM), 10 μL of 2 N TaqMan Universal PCR Master Mix No Amp Erase UNG, and 5 μL of reverse‐transcribed product. The reaction was first incubated at 95°C for 10 min followed by 40 cycles at 95°C for 15 s and at 60°C for 1 min. The quantitative real‐time PCR (RT‐qPCR) was performed on an ABIPRISM 7500 Real Time PCR System. Data was analysed by a 7500‐system software (1 1.4.0) with the automatic comparative threshold (*Ct*) setting for adapting baseline. Detection thresholds were set at 35 *Ct*. The relative amounts of miR‐34 and miR‐124a were calculated using the *Ct* method: Δ*Ct* = *Ct* (miR‐34/miR‐124a) − *Ct* (reference miRNA); 2−Δ*Ct*.

### 2.6 Data Analysis

Data were expressed as mean (standard error, SE) or median (interquartile range, Q1–Q3) as appropriate. A repeated measures ANOVA test with ‘time’ as the within‐subject factor was used to evaluate the differences within the same subjects at the three different time points (T0, T1 and T2) and ‘group’ as the between‐subject factor to compare the two groups (Melatonin and Control groups) at each time point. The Friedman non‐parametric test followed by Dunn's test was used for SIRT1 levels. Linear regression analyses were also performed to examine correlations between variables (Pearson correlation). All data with *p* < 0.05 were considered statistically significant. GraphPad Prism 6.0 (GraphPad Software Inc., San Diego, CA, USA) was used for statistical analysis.

## 3 Results

### 3.1 Clinical Data

During the study period, 50 patients were screened for eligibility, 44 children who met eligibility criteria were randomised, and 22 for each of the two treatment conditions. Due to insufficient blood, pre‐analytical problems with samples, and withdrawal of consent, 31 patients were finally completely and longitudinally analysed. Table 1 reports the clinical characteristics of the enrolled population.

**TABLE 1:** Clinic characteristics of the enrolled population.

	Melatonin group (*n* = 19)	Control group (*n* = 12)	*p*
Age (years ± SE)	4.0 ± 0.1	3.9 ± 0.1	NS
Body weight (kg ± SE)	18.6 ± 0.3	17.6 ± 0.3	NS
Gender	2 (F) 17 (M)	1 (F) 11 (M)	NS
Cryptorchidism	10	7	—
Phimosis	5	2	—
Inguinal hernia	2	1	—
Hydrocele	2	2	—
Length of surgery (min ± SE)	35.3 ± 1.5	36.3 ± 2.2	NS
Paracetamol doses (*n* ± SE)	3.3 ± 0.1	4.2 ± 0.2	< 0.001

Children enrolled were comparable in terms of age, weight, type of surgical procedures and duration of anaesthesia. The distribution of female subjects was 2/19 and 1/12 in the melatonin‐treated and Control groups, respectively. The main reason for surgical treatment was cryptorchidism (Table 1).

### 3.2 Pre‐ and Post‐Surgery Melatonin Plasma Concentrations

**FIGURE 1:**
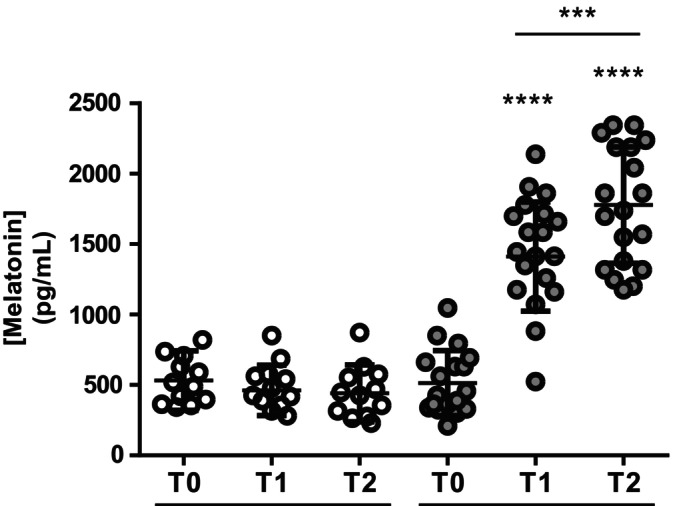
Pre‐ and post‐surgery plasma melatonin concentrations. Plasma melatonin concentration (pg/mL) evaluated at T0 (pre‐hospitalisation, 1 day before surgery), T1 (after anaesthesia, immediately before surgery) and T2 (at 1 h after the end of the surgery and awakening after anaesthesia) in untreated (Control, *N* = 12) and treated with melatonin (*N* = 19) children. ****p ≤ 0.0001 versus melatonin T0 and ***p ≤ 0.001 melatonin T2 versus melatonin T1 (Tukey's multiple comparisons test).

To investigate the variation of circulating melatonin in children undergoing surgery, melatonin plasma concentrations were assessed longitudinally in all patients in the pre‐ and postoperative periods. First, melatonin plasma concentrations were analysed in the preoperative period at T0 (pre‐hospitalisation, 1 day before surgery), T1 (after anaesthesia, immediately before surgery) and T2 (at 1 h after the end of the surgery and awakening after anaesthesia). As shown in Figure 1, plasma melatonin concentrations were not modulated by surgery in the untreated Control children. In contrast, significant differences were observed in melatonin‐treated infants (*F* = 63.7 and *p* < 0.0001 between T0, T1 and T2 by Repeated measures ANOVA). In detail, melatonin plasma concentrations significantly increased before surgery (T1) compared to those observed before hospitalisation (T0) (1521.4 ± 93.87 pg/mL (T1) vs. 554.9 ± 56.10 pg/mL (T0), *p* < 0.0001; Figure 1) and further increased after surgery (1892.1 ± 92.43 pg/mL (T2) vs. 1521.4 ± 93.87 pg/mL (T1), *p* < 0.001; Figure 1).

### 3.3 Melatonin Supplementation Reduces 4‐HNE Plasma Concentrations

**FIGURE 2:**
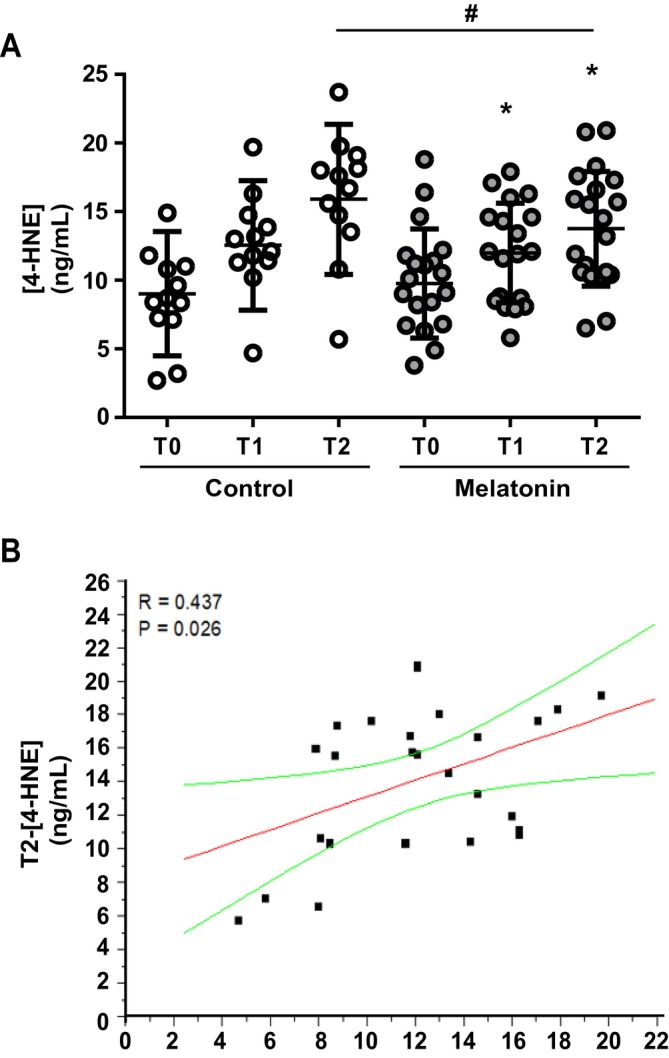
Pre‐anaesthesia and post‐surgery plasma 4‐HNE concentrations. (A) Plasma 4‐HNE concentration (ng/mL) evaluated at T0 (pre‐hospitalisation, 1 day before surgery), T1 (after anaesthesia, immediately before surgery) and T2 (at 1 h after the end of the surgery and awakening after anaesthesia) in untreated (Control, *N* = 12) and treated with melatonin (*N* = 19) children. *p ≤ 0.05 versus melatonin T0 and ^#^ *p* ≤ 0.05 melatonin T2 versus Control T2 (Tukey's multiple comparisons test). (B) Correlation between 4‐HNE plasma levels at T1 and T2.

**FIGURE 3:**
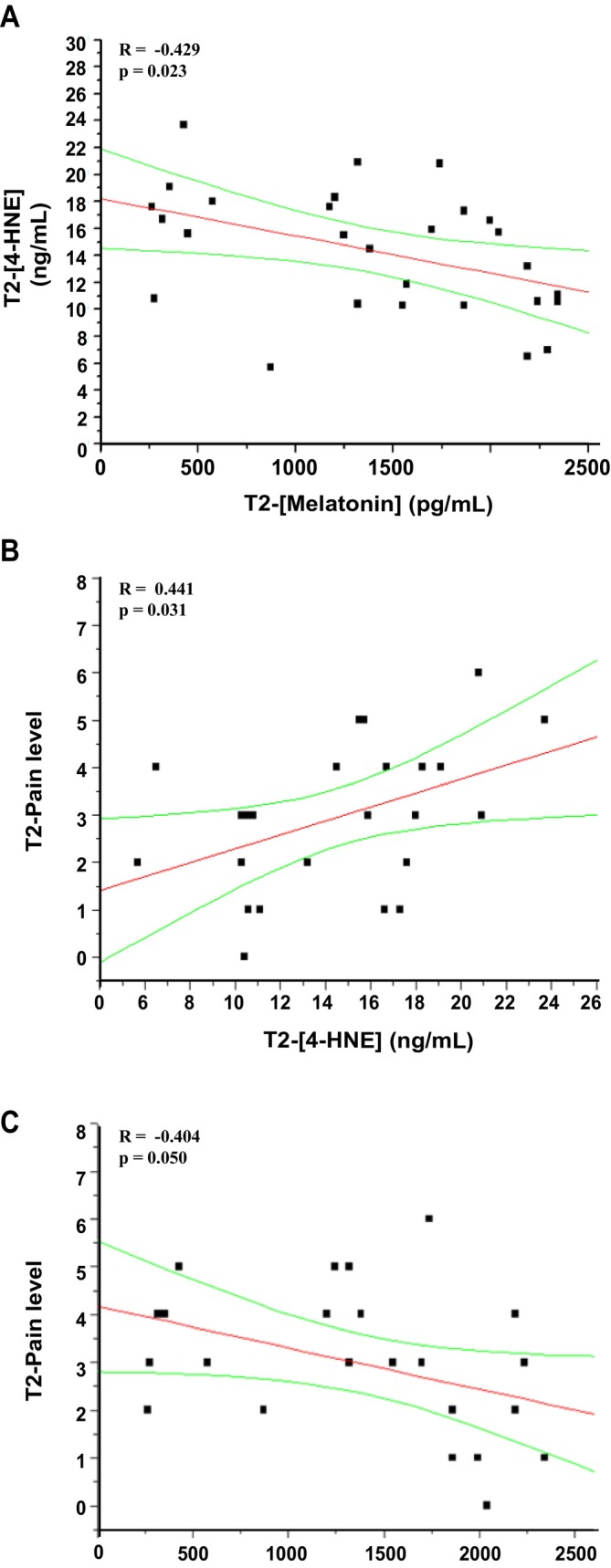
Correlations between melatonin, 4‐HNE and pain levels at T2. Linear regression analysis between (A) melatonin and 4‐HNE plasma levels, (B) 4‐HNE plasma levels and pain levels and (C) melatonin plasma levels and pain levels evaluated at T2 (at 1 h after the end of the surgery and awakening after anaesthesia) in untreated (*N* = 12) and treated with melatonin (*N* = 19) children.

The mean blood concentration of 4‐HNE in the Control group, although not reaching statistical significance due to the limited number of samples analysed, showed an increase from the pre‐ to the postoperative period (Figure 3A; 9.2 ± 2.27 ng/mL, 12.5 ± 1.78 ng/mL, 15.9 ± 1.93 ng/mL at T0, T1 and T2, respectively), suggesting an increase in concentration due to surgery. A similar statistically significant trend was observed in infants treated with melatonin (*F* = 7.704 and *p* = 0.0042 by repeated measures ANOVA), presenting 4‐HNE values equal to 9.8 ± 0.99 ng/mL, 11.6 ± 0.83 ng/mL and 14.0 ± 0.97 ng/mL at T0, T1 and T2, respectively (*p* < 0.05, T1 vs. T0; *p* < 0.05 T2 vs. T0, Figure 2A). Melatonin supplementation significantly reduced 4‐HNE plasma levels compared to the Control group at awakening (*p* < 0.05 Melatonin T2 vs. Control T2, Figure 2A). The increasing effect of surgery on 4‐HNE levels was strongly confirmed by the positive correlation observed between the levels of the lipid peroxidation markers at T1 and T2 (*R* = 0.437, *p* = 0.026, Figure 2B). Furthermore, melatonin and 4‐HNE plasma levels showed a clear negative correlation at the time of awakening after anaesthesia (*R* = −0.429, *p* = 0.023, Figure 3A). Interestingly, the increased 4‐HNE plasma levels observed at T2 were positively correlated with high pain scores (*R* = 0.441, *p* = 0.031, Figure 3B), while increased melatonin concentrations were associated with lower pain scores (*R* = −0.404, *p* = 0.050, Figure 3C).

### 3.4 Melatonin Supplementation Increases SIRT1 Plasma Concentrations

**FIGURE 4:**
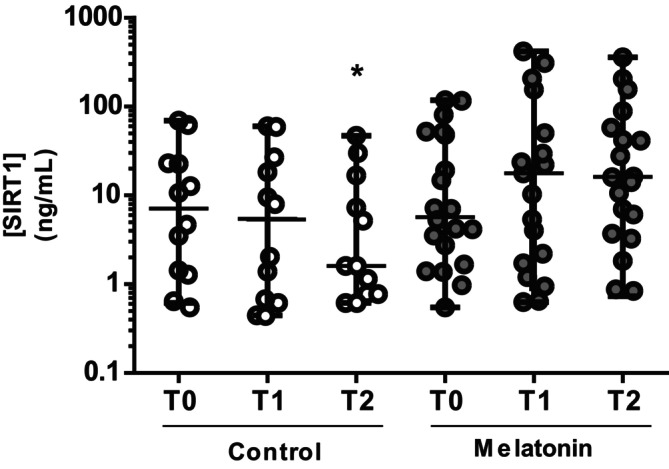
Pre‐ and post‐surgery plasma SIRT1 concentrations. Plasma SIRT1 concentration (ng/mL) was evaluated at T0 (pre‐hospitalisation, 1 day before surgery), T1 (after anaesthesia, immediately before surgery) and T2 (at 1 h after the end of the surgery and awakening after anaesthesia) in untreated (Control, *N* = 12) and treated with melatonin (*N* = 19) children. *p < 0.05 versus Control T0 (Dunn's multiple comparisons test).

**FIGURE 5:**
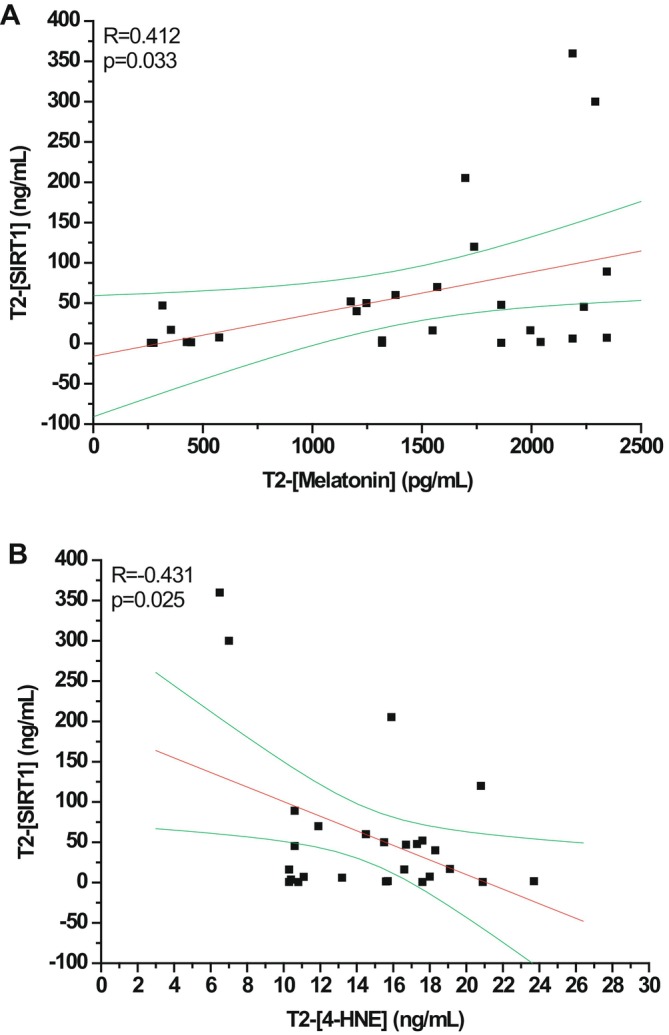
Correlations between melatonin, 4‐HNE and SIRT1 levels at T2. Linear regression analysis between (A) melatonin and SIRT1 levels and (B) 4‐HNE and SIRT1 levels evaluated at T2 (at 1 h after the end of the surgery and awakening after anaesthesia) in untreated (*N* = 12) and treated melatonin (*N* = 19) children.

Plasma SIRT‐1 concentrations were significantly modulated by surgery in untreated Control children (*p* = 0.046 by Friedman test). Indeed, SIRT1 levels (expressed as median [IQR]) showed a significant decrease from the pre‐ to the postoperative period (Figure 4; 7.10 (1.23–34.4) ng/mL, 5.43 (0.57–28.7) ng/mL, 1.60 (0.78–18.8) ng/mL at T0, T1 and T2, respectively; *p* < 0.05 T2 vs. T0), suggesting a decrease in concentration due to surgery. SIRT1 plasma levels in the melatonin‐treated group, although not reaching statistical significance, showed a trend in increase from T0 to T1 and T2 (Figure 4; 5.68 (1.47–51.3) ng/mL, 17.7 (1.69–103.8) ng/mL, 16.2 (3.24–61.4) ng/mL at T0, T1 and T2, respectively). The increasing effect of melatonin on SIRT1 levels was confirmed by the positive correlation observed between the levels of the two markers at T2 (*R* = 0.412, *p* = 0.033, Figure 5A). Furthermore, SIRT1 and 4‐HNE plasma levels showed a clear negative correlation at the postoperative time point (*R* = −0.431, *p* = 0.025, Figure 5B).

### 3.5 Melatonin Supplementation Modulates Circulating Levels of miR‐34 and miR‐124a

**FIGURE 6:**
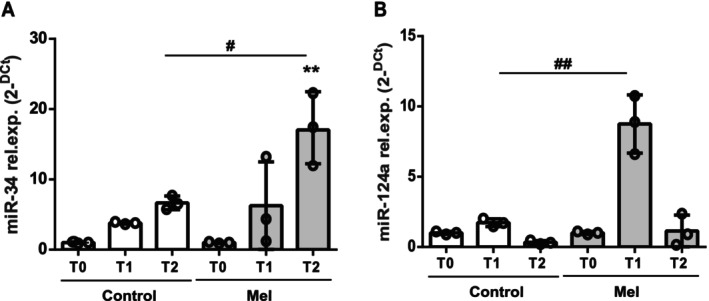
Pre‐ and post‐surgery circulating levels of miR34 and miR124a. Relative expression analysis of circulating miR‐34 (A) and miR‐124a (B) evaluated at T0 (pre‐hospitalisation, 1 day before surgery), T1 (after anaesthesia, immediately before surgery) and T2 (at 1 h after the end of the surgery and awakening after anaesthesia) in untreated (Control) and treated with melatonin children. *N* = 3 at each time point. **p ≤ 0.01 versus melatonin T0, "^#^ *p* ≤ 0.05 melatonin T2 versus Control T2 and ^##"^ *p* ≤ 0.01 melatonin T1 versus Control T1 (Tukey's multiple comparisons test).

We analysed some circulating microRNAs (miRNAs) targeting SIRT1, that is, miR‐34 and miR‐124a [16] (Figure 6). As shown in Figure 6A, the Melatonin group showed significantly higher circulating miR‐34 levels compared to the Control group at T2, that is, 1 h after the end of the surgery and awakening after anaesthesia (*p* < 0.05, Figure 6A). Circulating miR‐124a levels were not affected in Control infants, whereas a significant increase was observed at T1 (after anaesthesia, immediately before surgery) in melatonin‐treated children compared to the Control group (*p* < 0.01, Figure 6B).

## 4 Discussion and Conclusions

Preclinical and clinical studies have shown the efficacy of melatonin in different pain paradigms, but several questions related to the mechanism of action remain unclear [19, 20, 21]. For example, exogenous melatonin is known to induce anti‐nociception through the interaction of melatonin receptors, opioid receptors, NO, and NMDA systems. However, it is necessary to do additional research to know how endogenous melatonin modulates pain.

This study tests the hypothesis that melatonin reduces pain and oxidative stress involving the SIRT1 pathway. Clinical assessment of pain and biomarkers of OS was evaluated during the perioperative period in children undergoing abdominal surgery. We found a strong relationship between pain and 4‐HNE and between 4‐HNE and melatonin levels. The higher the melatonin, the lower the 4‐HNE; the lower the 4‐HNE, the lower the pain score.

Plasma levels of 4‐HNE are good indices of free radical damage to lipids. Lipid damage from free radical exposure leads to lipid hydroperoxide generation from lipids and to carbonyl formation and protein hydroperoxide generation from proteins [22]. The lipid peroxidation reaction leads to increased endogenous production of reactive aldehydes and their derivatives, such as malondialdehyde and 4‐HNE [23]. Malondialdehyde is the most mutagenic by‐product of lipid peroxidation, while 4‐HNE is the most toxic. 4‐HNE is often used as a marker of OS and has been implicated in the pathogenesis of cancer, neurodegenerative diseases, diabetes and other diseases [24]. 4‐HNE is involved in many pathophysiological signalling pathways; it has been found that 4‐HNE leads to the activation of NF‐κB, Nrf2, and mTOR pathways and inhibits the activity of the AKT/PKB pathway [25]. Furthermore, several studies have shown that lipid peroxidation is involved in different types of pain, such as cancer pain, fibromyalgia and neuropathic pain [26, 27, 28].

The significant reduction of 4‐HNE after melatonin administration in the postoperative period reflects the antioxidant properties of melatonin in vivo, allowing qualitative analysis of postoperative stress and guiding the clinical management of children undergoing surgery [29, 30].

The beneficial effects of exogenous melatonin supplementation in infants and children have been previously shown, indicating that its antioxidant action reduces serum inflammatory parameters and improves the clinical course of infants undergoing surgery [31, 32, 33]. Melatonin may play a key role in inhibiting the inflammatory cascade activated by surgical trauma, also known as the acute phase response [13]. Melatonin directly controls OS status as a radical scavenger, indirectly promotes the activity of antioxidant enzymes, inhibits pro‐oxidant enzymes, and reduces the toxicity of numerous drugs [34].

Focusing on perioperative pain, it was found that the increase in melatonin concentrations and the reduction in OS was significantly associated with low pain scores. This observation highlights the link between OS and pain [10, 35] and the clinical value of melatonin in paediatric surgery.

Interestingly, these melatonin effects parallel modulation of the circulating SIRT1 levels, which significantly decreased during surgery in the Control group, while showing a clear increase in melatonin‐treated patients. SIRT1, acting as a NAD^+^‐dependent deacetylase, plays a crucial role in critical cellular functions such as energy metabolism, genomic stability, inflammation and immune response [36]. Due to its multifunctional role, SIRT1 has been considered a promising target for therapies aimed at treating various conditions, including metabolic disorders and age‐related diseases [36]. Decreased SIRT1 levels have been observed in the spinal cord of various pathological pain models [37, 38] in which SIRT1 activation helped alleviate chronic pain by regulating inflammation, oxidative stress and mitochondrial dysfunction [14]. Here, we found that circulating SIRT1 was affected by surgery and preserved by melatonin, leading to OS reduction and a low requirement for pain treatment.

Recent animal studies have demonstrated that miRNAs are involved in pain processing pathways [39]. Ikuma et al. found the increased extracellular release of microRNAs from dorsal root ganglion cells in a rat model of neuropathic pain [40]. Here, we report preliminary data showing increased plasma levels of miR‐34a and miR‐124a in the melatonin‐treated group compared to control patients, suggesting a potential effect of melatonin on circulating microRNA expression. The observed upregulation of circulating miR‐124a and miR‐34a expressions in melatonin‐treated children could be explained by the following: studies have proposed that miR‐124a and miR‐34a exhibit an anti‐inflammatory effect by targeting the PIK3/NF‐kB pathway [41]. Moreover, it was hypothesised that overexpression of miR‐124a and miR‐34a stimulates G1‐phase cell‐cycle arrest and inhibits the production of matrix metalloproteinase and pro‐inflammatory cytokines (IL‐6, TNFα) [42]. In our study, miR‐124a and miR‐34a were associated with increased SIRT1 levels, likely alleviating the OS‐mediated pain. Notably, it was observed that miRNAs may not always follow a simple inverse relationship with their target proteins [43]. The increased miRNA levels observed in our study could reflect a compensatory response or an adaptive mechanism, possibly triggered by the initial postoperative inflammation or OS [44]. Melatonin, indeed, influences not only the expression of miRNAs but also the activity or stability of SIRT1, leading to higher protein levels despite elevated miRNA expression.

Overall, these findings demonstrate, for the first time, that melatonin significantly modulates SIRT1 circulating levels, reducing postoperative OS‐mediated pain, in a cohort of paediatric patients undergoing surgery, also involving miRNAs modulation. The study suggests a potential clinical application of melatonin for managing pain in postoperative paediatric surgery, possibly reducing reliance on traditional analgesics like paracetamol.

### 4.1 Strengths and Limitations

This study has some limitations. First, it is a pilot study with a relatively small sample size. Second, the study population consists of children aged 3–5 years undergoing elective surgery, which may limit the generalisability of the findings to other age groups. Third, downstream effects on nociceptive signalling and inflammation were not examined. Additionally, since we only had access to plasma samples, our ability to conduct more detailed cellular and molecular analyses was restricted. Future research could benefit from cellular and molecular studies to explore the underlying mechanisms of action. Thus, our findings should be considered hypothesis‐generating. Nonetheless, the study provides new insights into the modulation of key microRNAs by melatonin, which target SIRT1 activity, offering a potential novel pathway for melatonin's effects.

## Author Contributions

**Serafina Perrone:** conceptualization (lead), project administration (equal), supervision (equal), writing – original draft (equal), writing – review and editing (lead). **Silvia Carloni:** methodology (lead), supervision (equal), writing – original draft (lead). **Serena Benedetti:** investigation (lead), methodology (equal). **Micheal Weiss:** methodology (equal), supervision (equal), writing – review and editing (equal). **Lucia Marseglia:** investigation (equal), methodology (equal). **Valentina Dell'Orto:** data curation (supporting), writing – review and editing (equal). **Virginia Beretta:** data curation (equal), writing – review and editing (equal). **Noemi Pappagallo:** investigation (equal). **Marica Pagliarini:** methodology (equal). **Maria Cristina Albertini:** validation (supporting), writing – review and editing (equal). **Patrizia Ambrogini:** methodology (equal). **Walter Balduini:** validation (lead), writing – review and editing (supporting). **Angela Simona Montalto:** investigation (equal), writing – review and editing (equal). **Pietro Impellizzeri:** investigation (equal), writing – review and editing (equal). **Giuseppe Buonocore:** supervision (equal), validation (lead), writing – review and editing (equal). **Eloisa Gitto:** conceptualization (equal), project administration (lead), supervision (equal), writing – review and editing (equal). **Carmelo Romeo:** conceptualization (equal), investigation (equal), project administration (equal).

## Ethics Statement

The study was registered and approved by the local institutional ethics committee (ethics committee n 125222022) in accordance with the Helsinki Declaration of 1964.

## Consent

Written informed consent was obtained from the patient's legal guardians.

## Conflicts of Interest

The authors declare no conflicts of interest.

## Supporting information


**Figure S1.** CONSORT flow diagram, illustrating the allocation of participants in the study.

## Data Availability

Lead contact: Further information and requests for resources and reagents should be directed to and will be fulfilled by the lead contact, Serafina Perrone (saraspv@yahoo.it). Materials availability: This study did not generate new unique reagents. Data and code availability: All data reported in this paper will be shared by the lead contact upon request. This paper does not report the original code. Any additional information required to reanalyze the data reported in this paper is available from the lead contact upon request.
