# Supplementary figures and images for: Antioxidant and Analgesic Effect of Melatonin Involving Sirtuin 1: A Randomised Pilot Clinical Study
Source: J Cell Mol Med. 2025 May 19;29(10):e70605. doi: 10.1111/jcmm.70605 (PMC12087297; doi:10.1111/jcmm.70605)

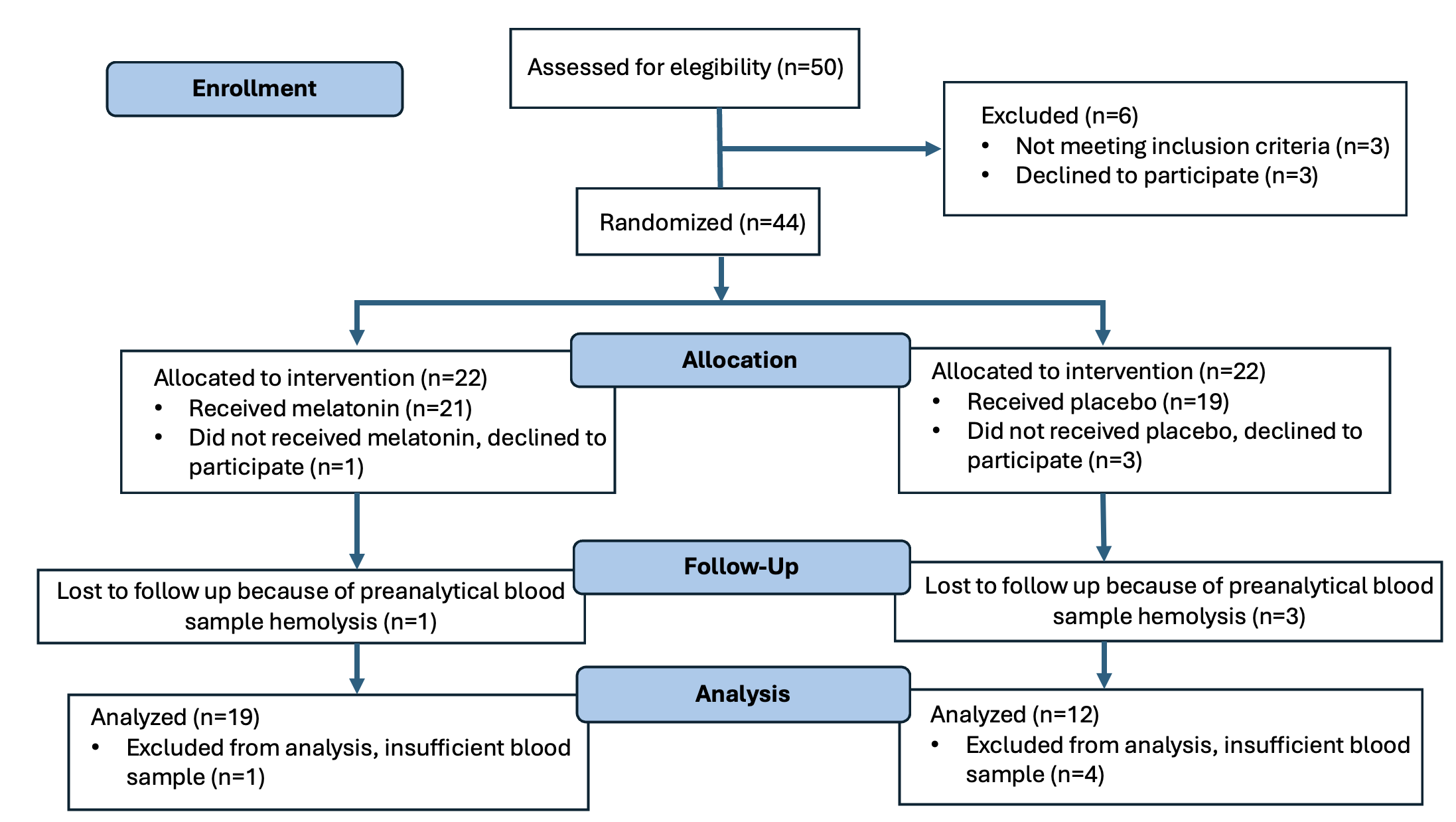

Supplement: Supplementary file 1 — Figure S1. CONSORT flow diagram, illustrating the allocation of participants in the study. [file JCMM-29-e70605-s001.png]
